# Lymphatic Regeneration after Popliteal Lymph Node Excision and Implantation of Aligned Nanofibrillar Collagen Scaffolds: An Experimental Rabbit Model

**DOI:** 10.3390/jfb15080235

**Published:** 2024-08-21

**Authors:** José Luis Campos, Gemma Pons, Ali M. Al-Sakkaf, Irene Laura Lusetti, Laura Pires, Francisco Javier Vela, Elena Ramos, Verónica Crisóstomo, Francisco Miguel Sánchez-Margallo, Elena Abellán, Jaume Masiá

**Affiliations:** 1 Department of Microsurgery, Jesús Usón Minimally Invasive Surgery Centre, 10071 Cáceres, Spain; lcpires@ccmijesususon.com (L.P.); fjvela@ccmijesususon.com (F.J.V.); eramos@ccmijesususon.com (E.R.); eabellan@ccmijesususon.com (E.A.); 2 Department of Plastic Surgery, Hospital de la Santa Creu i Sant Pau, 08025 Barcelona, Spain; gponsp@santpau.cat (G.P.); sakaf_250@yahoo.com (A.M.A.-S.); irenelaura.lusetti@gmail.com (I.L.L.); jmasia@santpau.cat (J.M.); 3 Jesús Usón Minimally Invasive Surgery Centre, 10071 Cáceres, Spain; crisosto@ccmijesususon.com (V.C.); msanchez@ccmijesususon.com (F.M.S.-M.); 4 Centro de Investigación Biomédica en Red de Enfermedades Cardiovasculares (CIBERCV), Instituto de Salud Carlos III, 28029 Madrid, Spain

**Keywords:** BioBridge, lymphedema, microsurgery, rabbit animal model, lymphatic regeneration

## Abstract

Lymphedema presents significant challenges to patients’ quality of life, prompting the exploration of innovative treatments, such as collagen scaffolds, aimed at treating and reducing the risk of lymphedema. We aimed to evaluate the preventive and therapeutic efficacy and the lymphangiogenic potential of implanted aligned nanofibrillar collagen scaffolds (BioBridge^TM^) following the induction of secondary lymphedema in a rabbit model. Thirty rabbits were divided into treatment (G1), prevention (G2), and control (G3) groups. Secondary lymphedema was induced in all groups. BioBridge^TM^ implantation was performed in G2 and G1 on days 0 and 60, respectively. Follow-ups included hindlimb circumference measurements and indocyanine green lymphography at 0, 60, and 90 days. None of the study rabbits exhibited dermal backflow on day 0 before surgery. At 60 days, the incidence rates of dermal backflow in G1, G2, and G3 were 100%, 44.4%, and 90%, respectively. Furthermore, at 90 days, the incidence rates were 22.2%, 44.4%, and 90%, respectively. New linear lymphatic observation was seen in rabbits with resolved dermal backflow. The findings of this study demonstrated the capacity of BioBridge^TM^ scaffolds to induce new lymphatic vessel formation and reduce dermal backflow in secondary lymphedema in a rabbit model.

## 1. Introduction

Lymphedema is a chronic condition that often results from cancer treatment, trauma, or parasitic infections that cause damage to lymphatic channels [1,2]. This condition manifests as a progressive pathological state in the lymphatic system, characterized by the interstitial accumulation of protein-rich fluid, inflammation, hypertrophy of adipose tissue, and fibrosis [1,3]. Secondary lymphedema is an acquired condition, linked to specific circumstances or treatments, including surgery for malignant tumors involving lymph node dissection, inflammatory disorders, radiotherapy, infections, burns, trauma, obesity, and chronic venous insufficiency [4]. The incidence of lymphedema in the upper extremities among women post-mastectomy is reported to be 24–49%, and its onset may occur within a few days or up to 30 years following treatment [5,6]. In contrast, lower extremity lymphedema is observed in patients treated for uterine or prostate cancer, lymphoma, or melanoma [4].

Over the past decade, advances in microsurgical techniques and studies involving animal models have motivated a deeper exploration of lymphedema pathophysiology, introducing novel perspectives and enhancing diagnostic and therapeutic methodologies [1,7]. Conventional lymphedema treatment adopts a palliative approach to reduce swelling and manage infection. Conservative interventions encompass techniques such as massage and compression garments intended to facilitate lymphatic fluid drainage and alleviate its accumulation thereof [8,9]. Additionally, microsurgical strategies such as vascularized lymph node transfer (VLNT) [10] and lymphovenous anastomosis (LVA) [11] are employed to treat patients with symptomatic secondary lymphedema. However, mastering these microsurgical techniques presents a steep learning curve because of the challenges in identifying and manipulating lymphatic vessels [12]. Moreover, their volume reduction rates fall below 60% after surgery, necessitating further refinement of these procedures [13,14,15].

Contemporary investigations have aimed to identify safer options to reconstruct the impaired and obstructed lymphatic ducts in patients with lymphedema. This involves the synergistic application of microsurgical techniques, advanced imaging technologies [16,17,18,19], and innovative scaffolds like BioBridge^TM^ [2].

Fibralign Corp has pioneered the creation of an aligned nanofibrillar collagen scaffold known as BioBridge^TM^, designed to replicate the collagen matrix found in vascular structures, to investigate lymphatic regeneration [2]. This scaffold features a multi-lumen structure, which, upon implantation, induces a unidirectional interstitial capillary flow propelled by hydrostatic and oncotic pressure [2]. Preliminary investigations indicate that BioBridge^TM^ effectively directs cell organization, regulates endothelial inflammatory responses, and enhances cell survival following implantation in both normal and ischemic tissues [20,21,22,23,24].

To investigate the prevention, treatment, and application of microsurgical techniques for lymphedema, it is imperative to establish an animal model in which chronic diseases can be induced and remain stable over an extended period [25,26]. In this study, we used a pre-established model of secondary lymphedema in rabbit hindlimbs. The anatomical features of rabbits, especially their body proportions, facilitate the straightforward identification of the lymphatic vasculature and popliteal lymph nodes (PLN) in the hindlimb. This allows relatively uncomplicated access to these structures through a simple surgical procedure [27,28,29]. We, therefore, aimed to assess the preventive and treatment efficacy of BioBridge^TM^ implantation following the excision of the PLN and sectioning of the afferent lymphatic vessel of the inguinal lymph node (ILN) in the left hindlimbs of rabbits, with a specific focus on assessing its capacity to stimulate lymphangiogenesis.

## 2. Materials and Methods

This study was conducted at the Jesús Usón Minimally Invasive Surgery Center, J.U.M.I.S.C. (Cáceres, Spain), after obtaining approval from the institutional review board. All parameters adhered to the guidelines for quantifying pain, stress, and distress in laboratory animals [30]. The protocol, including any amendments or procedures related to the care or use of animals, underwent a thorough review and approval by the Testing Facility Institutional Animal Care and Use Committee and the Government of the Junta de Extremadura (EXP-20230312) in accordance with European legislation before the initiation of such procedures.

This study aimed to assess the preventive and therapeutic efficacy of BioBridge (Fibralign Corp., Union City, CA, USA) implantation and its potential to induce lymphangiogenesis in an animal model. The study utilized a cohort of 30 healthy 1-year-old female rabbits (New Zealand White rabbits; Granja San Bernardo, Tulebras, Navarra, Spain), and surgical procedures were conducted on the left hindlimb using the right hindlimb as a control.

The study population was randomly divided into three groups of 10 rabbits each using Microsoft^®^ Excel^®^ Microsoft 365 MSO (Version 2207 Build 16.0.15427.20248) as follows: a treatment group (G1), a preventive group (G2), and a control group (G3). Indocyanine green (ICG) lymphography was used to delineate the lymphatic vessels in the hindlimbs, PLN, and ILN. Each rabbit was subjected to digital video recording, facilitated by a camera equipped with an infrared filter (Fluobeam; Fluoptics; Grenoble, France), to capture and document ICG contrast (25 mg; Verdye; Diagnostic Green Limited, Westmeath, Ireland).

Intraoperatively, Patent Blue V (PBV) (2.5 g/100 mL; Bleu Patente V Sodique; Guerbet, Villepinte, France) was used to visualize the lymph nodes and lymphatic vessels in the popliteal fossa and inguinal area. Follow-up, euthanasia, and left hindlimb sample collection were conducted at specific time points (0–30 and 60 days post-surgery). Two independent researchers were involved in the data collection.

### 2.1. Pre-Operative Procedures

The co-induction phase of anesthesia was initiated by administering midazolam (5 mg/mL; Normon S.A, Madrid, Spain) at a dose of 0.5 mg/kg and propofol (10 mg/mL; Propomitor; Orion Pharma, Spoo, Finland) at a dose of 10 mg/kg via infusion into the ear marginal vein. All rabbits were subsequently intubated using 3.0–3.5 endotracheal tubes connected to a semi-closed circuit, maintaining sevoflurane (1000 mg/g; SevoFlo; Zoetis Belgium, Luvain-la-Neuve, Belgium) at a concentration of 3.9–4.5%. During the intra-operative phase, analgesia was achieved through the administration of ketorolac (30 mg/mL; Normon S.A, Madrid, Spain) at a dose of 1.5 mg/kg in combination with tramadol (50 mg/mL; Normon S.A, Madrid, Spain) at a dose of 3 mg/kg.

**Figure 1:**
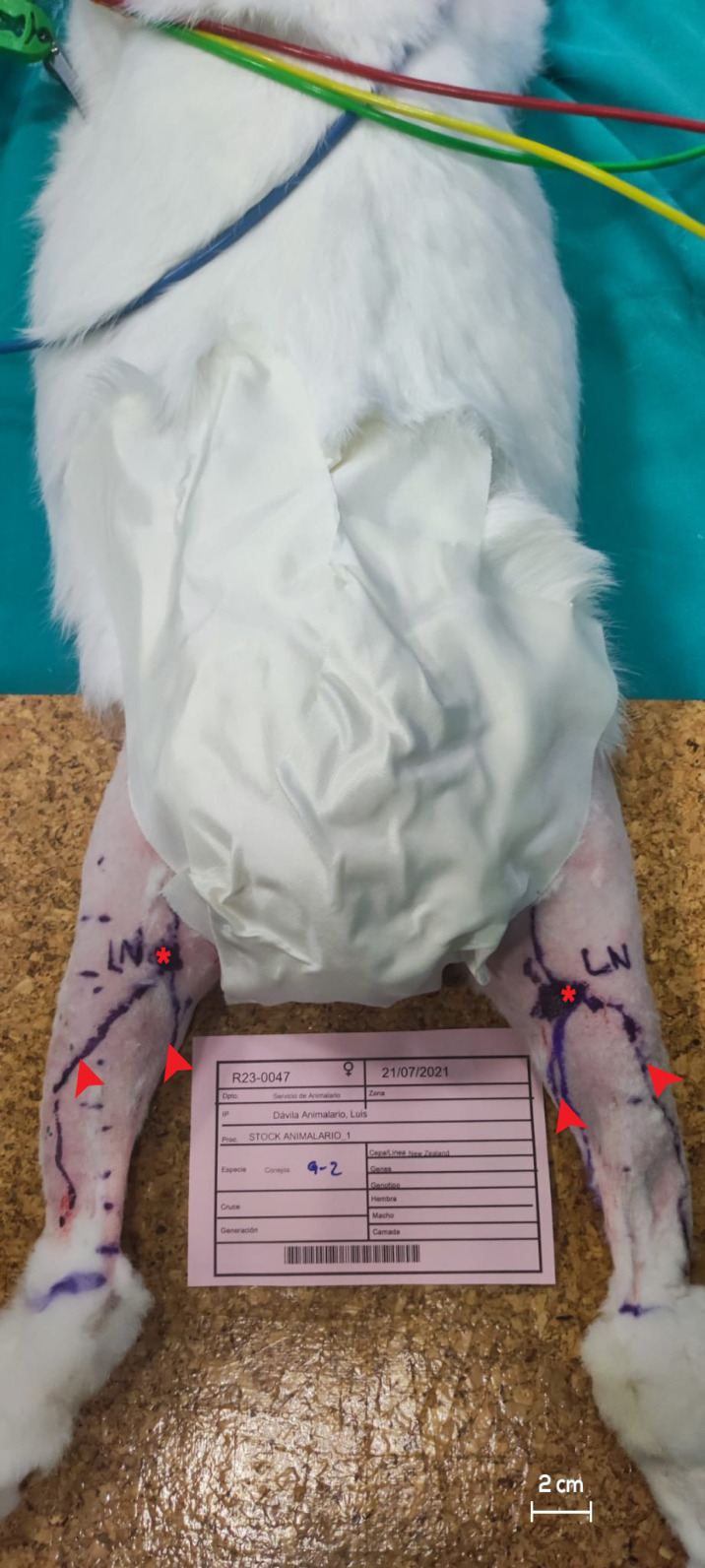
Pre-operative observations in a rabbit model before PLN excision and ILN sectioning. The viability of the popliteal lymph node (red asterisks) and hindlimb lymphatic vessels (red arrowheads) was confirmed via ICG lymphography, and they were labeled using a surgical marker. For all figures, indocyanine green (ICG) was used. PLN, popliteal lymph node. ILN, inguinal lymph node.

Prior to the surgical procedures, the study subjects underwent thorough depilation of their hindlimbs up to the inguinal region, followed by skin antisepsis. The rabbits were positioned in the prone orientation for excision of the PLN and subsequently in the supine orientation for sectioning of the afferent lymphatic vessels of the ILN (Figure 1).

### 2.2. Intra-Operative Procedures

**Figure 2:**
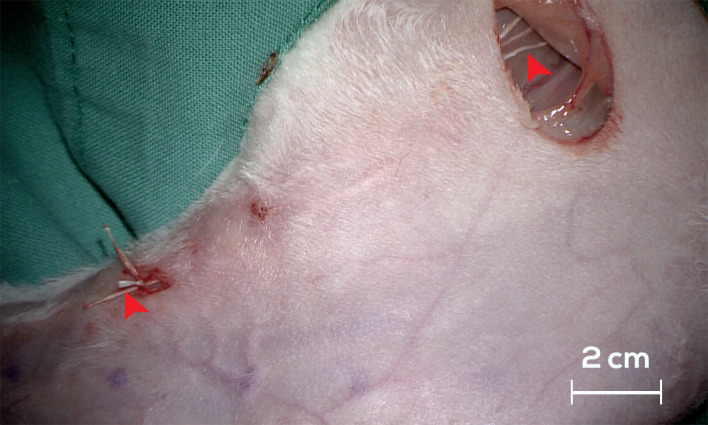
The surgical technique and placement of the BioBridge^TM^ device from the ILN (red arrowhead on the right side) to the PLN (red arrowhead on the left side).

To visualize the vessels and lymph nodes, 0.2 mL of PBV was intradermally administered into the interdigital spaces of the left hindlimb. Following the injection, the region underwent gentle massage, and controlled flexion and extension movements of the hindlimb were performed for a few minutes to enhance the absorption of the dye into the lymphatic vessels. On day 0, an open surgical approach with a 2 cm transverse incision was employed in the popliteal fossa area following the administration of ICG and PBV to identify the PLN. Subsequently, PLN excision was performed in all three groups, cutting the afferent lymphatic vessels and coagulating the efferent lymphatic vessels [28]. In the case of the ILN, vascular structures were identified using a similar open surgical approach with a 2 cm longitudinal incision. The afferent lymphatic vessels of the ILN were sectioned in all three study groups by coagulating the distal end and cutting the proximal end. After the surgical procedures, three BioBridge^TM^ scaffolds were subcutaneously implanted in G2, extending from the ILN to 4 cm below the PLN. A 60-day latency period (transit phase) was implemented to induce lymphedema in the left hindlimbs of all the groups. Subsequently, three BioBridge^TM^ scaffolds were subcutaneously implanted in G1 following a previously described procedure (Figure 2).

### 2.3. Postoperative Procedures

In the postoperative period, buprenorphine (300 μg/mL; Bupaq; Richter Pharma, Wels, Austria) was administered at a dose of 30 mg/kg, along with meloxicam (5 mg/mL; Meloxidyl; Ceva Santé Animale, Libourne, France) at a dose of 2 mg/kg and enrofloxacin (10 mg/mL; Baytril; Bayer Animal Health Gmbh, Leverkusen, Germany) at a dose of 5 mg/kg, continued for 5 days post-surgery to prevent infection. The same anesthetic protocol as described previously was employed during the 30-day and 60-day postoperative follow-up assessments. Euthanasia was performed on day 60 using intravenous potassium chloride (20 mmol/10 mL; B. Braun, Barcelona, Spain) administered at an average rate of 2 mEq/kg into the auricular vein.

**Figure 3:**
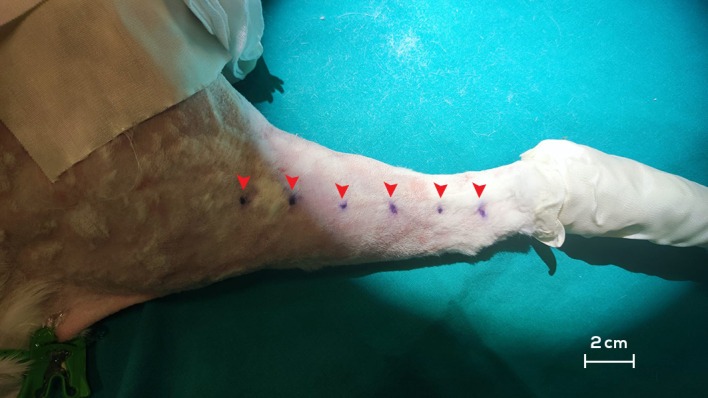
Measurement of the hindlimb volume of the rabbit at 2 cm intervals. The points delineated with a surgical marker correspond to points 0, 2, 4, 6, 8, and 10 (red arrowheads).

Follow-up assessments were conducted on both hindlimbs at postoperative intervals of 0, 60, and 90 days. These evaluations comprised circumferential measurements of each hindlimb at 2 cm intervals, starting from the distal aspect of the tarsus (measurement 0) to the distal aspect of the femur (measurement 10) (Figure 3); individual rabbit weight measurements; and ICG lymphography for comprehensive visualization of the lymphatic system and the detection of dermal backflow. Following the final collection of relevant data, hindlimb samples were harvested for subsequent histopathological and immunohistochemical analyses.

### 2.4. Histopathological and Immunohistochemical Analyses

Hematoxylin and eosin staining (H&E) was utilized to histologically examine the cutaneous and subcutaneous tissues of the hindlimb. Histological sections were digitally scanned using an Aperio GT 450 DX system (Leica Biosystems, Barcelona, Spain). The parameters assessed included the presence of edema and lymphangiogenesis. Additionally, immunohistochemical analysis was conducted to evaluate the lymphatic markers CD31 and podoplanin.

### 2.5. Statistical Analysis

The data are expressed as means ± standard error. Group differences were assessed using the Kruskal–Wallis and Mann–Whitney U tests, while intragroup comparisons were conducted using a Wilcoxon paired samples test. Statistical significance was set at *p* < 0.05. All *p*-values were derived using two-tailed tests. Statistical analyses were performed using the IBM SPSS Statistics 27 software for Windows.

## 3. Results

### 3.1. Treatment Group (G1)

**Figure 4:**
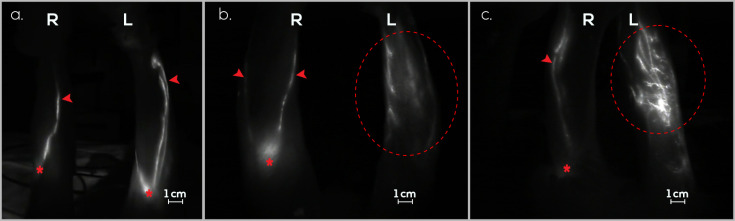
Monitoring lymphatic patency and assessing secondary lymphedema in the right (R) and left (L) hindlimbs of rabbits using ICG lymphography in the treatment group (G1). (**a**). Evaluation of both hindlimbs on day 0 before secondary lymphedema induction. The afferent lymphatic vessels (red arrow) and PLN (red asterisk) are visible. No dermal backflow is detected. (**b**). Assessment of both hindlimbs on day 30 after secondary lymphedema induction. The afferent lymphatic vessels (red arrow), PLN (red asterisk), and dermal reflux image in the left hindlimb (red dashed circle) are evident. (**c**). Examination of both hindlimbs on day 60 after secondary lymphedema induction. The afferent lymphatic vessels (red arrow), the PLN (red asterisk), and an image indicative of new lymphatic vessel formation in the left hindlimb (red dashed circle) are observed.

On day 0, 10 rabbits underwent PLN excision and afferent lymphatic vessel sectioning of the ILN in the left hindlimb, resulting in the death of one rabbit due to anesthesia complications. Following a 60-day interval for the induction of secondary lymphedema, 100% (9/9) of the rabbits exhibited dermal backflow in the hindlimb. Upon confirmation of edema through ICG lymphography, three BioBridge^TM^ devices spanning from the ILN to the PLN were implanted in each rabbit. Ninety days thereafter, dermal backflow was observed in 22.2% (2/9) of the rabbits. In rabbits without dermal backflow, new linear lymphatic observation was seen (Figure 4).

### 3.2. Preventive Group (G2)

**Figure 5:**
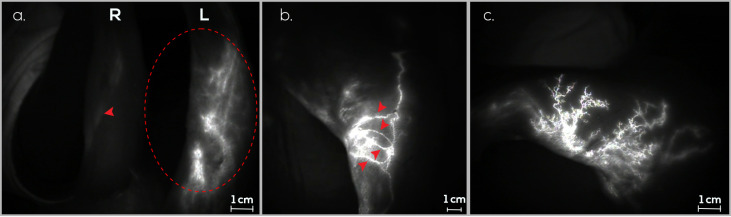
Monitoring lymphatic patency and assessing secondary lymphedema in the right (R) and left (L) hindlimbs of rabbits using ICG lymphography in the prevention group (G2). (**a**). Assessment of both hindlimbs on day 30 after secondary lymphedema induction and BioBridge^TM^ scaffold implantation. The presence of afferent lymphatic vessels (red arrow) and new lymphatic vessel formation in the left hindlimb (red dashed circle) is observed. (**b**). Evaluation of the inner thigh of the left hindlimb on day 60 after secondary lymphedema induction and BioBridge^TM^ scaffold implantation. New lymphatic vasculature formation is evident (red arrow). (**c**). Examination of the outer thigh of the left hindlimb on day 60 after secondary lymphedema induction and BioBridge^TM^ scaffold implantation. Lymphangiogenesis is evident, displaying a network of new lymphatic vessels.

On day 0, the rabbits underwent PLN excision, afferent lymphatic vessel sectioning from the ILN, and the implantation of three BioBridge^TM^ devices from the ILN to the PLN in the left hindlimb. One rabbit died within four days because of self-mutilation of the surgical wound. Following a 60-day assessment of secondary lymphedema formation using ICG lymphography, 44.4% (4/9) of the rabbits exhibited dermal backflow in the hindlimb. New linear lymphatic observation was seen in subjects without dermal backflow (Figure 5). This proportion remained consistent for 90 days after scaffold implantation.

### 3.3. Control Group (G3)

**Figure 6:**
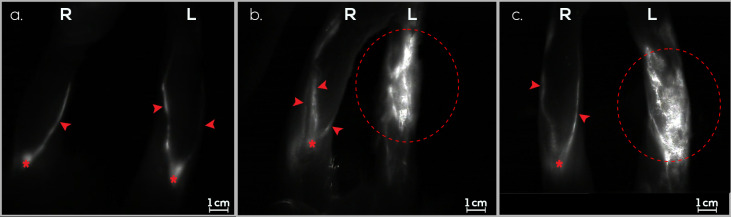
Monitoring lymphatic patency and assessing secondary lymphedema in the right (R) and left (L) hindlimbs of rabbits using ICG lymphography in the control group (G3). (**a**). Assessment of both hindlimbs on day 0 before secondary lymphedema induction. Afferent lymphatic vessels (red arrow) and PLN (red asterisk) are visible. No dermal backflow is detected. (**b**). Evaluation of both hindlimbs on day 30 after secondary lymphedema induction. Afferent lymphatic vessels (red arrow), PLN (red asterisk), and a dermal reflux image in the left hindlimb (red dashed circle) are evident. (**c**). Examination of both hindlimbs on day 60 after secondary lymphedema induction. Afferent lymphatic vessels (red arrow), PLN (red asterisk), and an image compatible with dermal reflux in the left hindlimb (red dashed circle) are observed.

On day 0, the rabbits underwent PLN excision and afferent lymphatic vessel sectioning from the ILN in the left hindlimb. Following a 60-day interval for the induction of secondary lymphedema, 90% (9/10) of the rabbits exhibited dermal backflow in the hindlimb, as confirmed by ICG lymphography. The incidence of secondary lymphedema remained constant until day 90 (Figure 6).

### 3.4. Follow-Up Assessments

**Figure 7:**
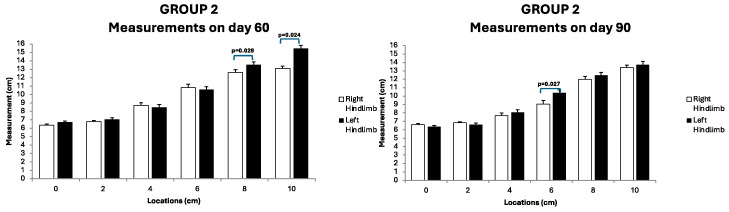
Circumferential measurements obtained on days 60 and 90 at all locations from the operated (**left**) and control (**right**) hindlimbs in rabbits belonging to G2.

**Figure 8:**
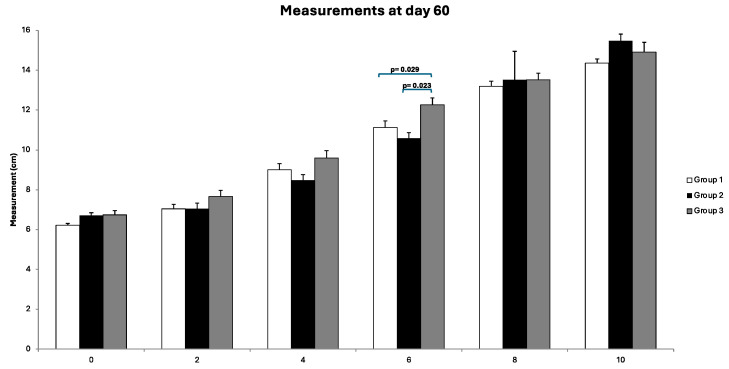
Circumferential measurements obtained in all groups on day 60 from the left hindlimb. Significant differences between the groups are seen at location 6.

Throughout the follow-up assessments, no anomalies in vital physiological parameters were observed. The mean weight of the rabbits was 4.8 kg (range, 3.7–6.2 kg). The mean diameter of the PLNs measured was 1.17 cm (range, 0.8–1.8 cm). Circumferential measurements of the right and left hindlimbs are shown in Figure 7. No significant differences were observed in any group when the treated hindlimb was compared to the control. However, the comparison of the diameters obtained in the operated hindlimbs among the three groups (assessed via the Kruskal–Wallis test) revealed significant differences only at location 6 after 60 days (*p* = 0.029). Post hoc tests indicated that these differences stemmed from significant variations between G1 and G3 and between G2 and G3 (Figure 8). After the completion of the follow-up period, the rabbits were euthanized according to the described protocol, and samples were collected for further analysis.

### 3.5. Histological Analysis

**Figure 9:**
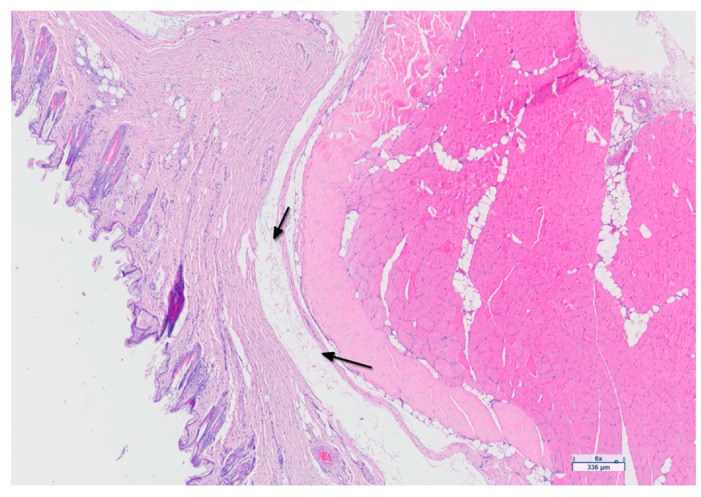
Digital microscopic image (Aperio GT 450 DX system) showing tissue structure separation attributable to edema (black arrows).

**Figure 10:**
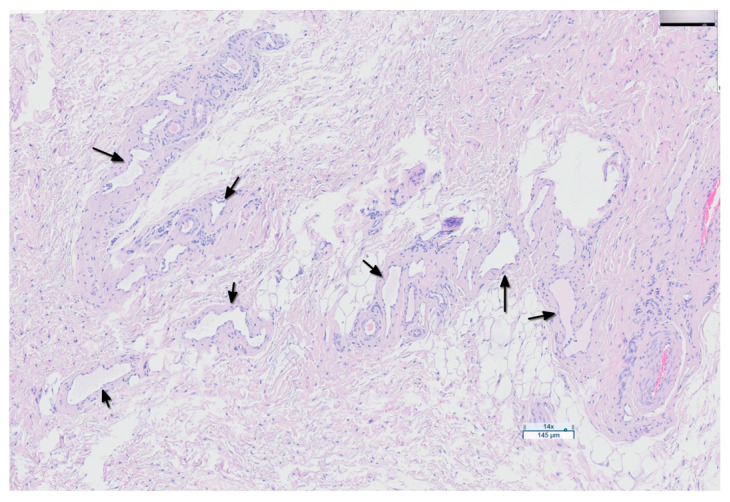
Digital microscopic image (Aperio GT 450 DX system) showing lymphangiogenesis in a specimen from a G1 study subject (black arrows).

A histological examination of the cutaneous and subcutaneous tissues of the hindlimbs was conducted to assess edema and lymphangiogenesis using routine staining (H&E) and immunohistochemical staining for the lymphatic markers CD31 and Podoplanin. The results revealed tissue structure separation consistent with secondary lymphedema in the hindlimbs of the G1 (22.2%, 2/9), G2 (44.4%, 4/9), and G3 (70%, 7/10) groups (Figure 9). Marked proliferation of the lymphatic vessels was observed in five rabbits from groups G1 and G2 treated with BioBridge^TM^, and in one rabbit from G3 (Figure 10). The results of the lymphatic marker analysis indicated adequate staining of the endothelium.

## 4. Discussion

None of the study rabbits exhibited dermal backflow on day 0 before surgery. At 60 days, the incidence rates of dermal backflow in G1, G2, and G3 were 100% (9/9), 44.4% (4/9), and 90% (9/10), respectively. Furthermore, at 90 days, the incidence rates were 22.2% (2/9), 44.4% (4/9), and 90% (9/10), respectively. The new linear lymphatic observation was seen in rabbits with resolved dermal backflow. Hindlimb circumference measurements showed no statistically significant differences between the treated and control hindlimbs. The histopathological results revealed lymphedema in the same proportion as that at the ICG follow-ups in G1 and G2, and in a smaller proportion of individuals in G3. The evaluation of tissue edema is inherently complex, as the serous edematous fluid is not easily discernible and is often removed during tissue processing. When edema is pronounced, it causes the separation of tissue structures, such as collagen fibers and muscle fibers, making it identifiable. However, when edema is mild, it can easily go unnoticed, rendering its assessment highly subjective. Lymphangiogenesis was strongly evident in five rabbits from G1 and G2 and in one rabbit from G3. Podoplanin was present in all subjects from G1 and G2 and in one subject from G3. CD31 was present in all the groups. These findings suggest that podoplanin exhibits greater sensitivity in identifying the presence of newly formed lymphatic vessels, although it does not achieve 100%. Conversely, regarding the lymphatic marker CD31, the data indicate its lack of sensitivity for lymphangiogenesis.

Currently, the sole therapeutic interventions available for individuals with lymphatic dysfunction include mechanical or manual lymphatic drainage, compression garments, or microsurgery [31]. Given that lymphangiogenesis involves the formation of new lymphatic vessels from pre-existing ones, occurring not only during development but also in adults, there is increasing interest in exploring engineered lymphatic vessels and scaffolds with functional attributes [24,32].

In this study, we employed an aligned nanofibrillar collagen scaffold (BioBridge^TM^), designed to emulate the collagen matrix of vascular structures, to examine lymphatic regeneration. Characterized by a multi-lumen structure, upon implantation, this scaffold induces a unidirectional interstitial capillary flow propelled by hydrostatic and oncotic pressure [2]. Preliminary investigations have demonstrated its capacity to orchestrate cellular organization, modulate the endothelial inflammatory response, and enhance cell survival by facilitating cell adhesion, proliferation, and migration along collagen fibrils post-implantation. This fosters the establishment of operational vessels within and surrounding the tissue-integrated scaffold in both normal and ischemic tissue contexts [20,21,33].

To develop effective management strategies for secondary lymphedema using this scaffold, it is imperative to establish a stable and reproducible animal model. Studies have illustrated the lymphangiogenic potential of BioBridge^TM^ following implantation in the hindlimbs of rat [2,23,34,35] and in pig models [22]. In the context of the rat model, its accessibility, cost-effectiveness, and ease of handling by researchers make it a favorable choice. Moreover, it anatomically and histologically recapitulates lymphedema in humans. However, a limitation of these animals is their small size, which renders the visualization of lymphatic structures more intricate and increases the risks associated with anesthesia. Additionally, their size precludes the undertaking of complex surgeries with prolonged follow-up times owing to potential postsurgical complications [36,37]. Concerning large animal models, such as pigs, researchers encounter substantial challenges related to management and cost. A notable limitation of the porcine hindlimb model is the lack of discernible macroscopic changes in the limb volume or circumference [38].

In this study, we opted for the rabbit model because it surpasses alternative choices, primarily because of its optimal average size, which is conducive to handling and housing. Additionally, rabbits offer a cost-effective solution and possess anatomical features that facilitate replicating surgical techniques employed in human participants [39]. Extended observation periods in these rabbits have revealed that the manifestation of hindlimb dermal backflow can persist consistently for 3–9 months following the induction of lymphedema [28]. Although no apparent increase in volume was observed in the lymphedematous hindlimb compared to its healthy counterpart in this model, the presence of secondary lymphedema was detectable by ICG lymphography [28]. This observation suggests that the rabbit hindlimb model effectively emulates the chronic progression characteristics of the disease in humans. Furthermore, postoperative follow-up can be conducted, as these procedures entail minimal risk to the rabbits’ lives and show remarkable healing capacity.

In our previous study, we demonstrated the suitability of this animal model for inducing secondary lymphedema through PLN excision and evaluated it using ICG lymphography [28]. However, a notable limitation was the small sample size. In the current study, we employed the same methodology, but addressed this limitation by expanding the number of rabbits and incorporating ILN sections to intensify the degree of dermal backflow. Notably, at the 60-day post-surgery mark, 90% (18/19) of the cases in G1 and G3 exhibited evident dermal backflow. In contrast, in G2, only 44.4% (4/9) presented with secondary lymphedema attributable to the preventive implantation of the BioBridge^TM^ device. Ninety days postoperatively, secondary lymphedema persisted in the left hindlimb in G2 and G3. However, in G1, only 22.2% (2/9) of the study subjects exhibited dermal backflow, with 77.8% (7/9) of the rabbits showing resolution of edema. This improvement was attributed to the prior implantation of the BioBridge^TM^ device on day 60. The outcomes observed in G1 and G2 underscore the lymphangiogenic capacity of this scaffold and its efficacy in resolving hindlimb edema in a rabbit model. These findings are consistent with those of analogous studies involving the use of this device in both human and animal models [2,22,24].

Several animal model studies in rats have shown a decrease in hindlimb volume after BioBridge^TM^ device implantation [2,23,34,35]. Conversely, in our study, measurements of rabbit hindlimb circumference revealed significant differences at location 6 after 60 days when comparing operated hindlimb diameters between G1 and G3, as well as between G2 and G3. However, no significant differences were observed in any group when the treated hindlimbs were compared with the control. These findings align with those of our previous study [28], suggesting that this assessment may not be the most essential part of future investigations into secondary lymphedema in rabbits because of the inability to replicate limb volume increases.

The histological findings of edema in G1 and G2 aligned with the data obtained using ICG lymphography to assess dermal backflow. However, the G3 results indicated a lower number of rabbits with lymphedema compared to those during the ICG follow-ups. This could be because serous edematous fluid is intangible and is removed during tissue processing. When mild, it can easily go unnoticed, and its histopathological assessment is highly subjective.

Regarding the lymphangiogenesis process, the results obtained regarding the appearance of neovessels in G1 and G2 were consistent with those obtained from the ICG follow-up. In G3, the proliferation of new lymphatic vessels was observed in only one rabbit, which may be attributed to the high regenerative capacity of this New Zealand white rabbit model.

The lymphatic markers used were podoplanin and CD31. Podoplanin is used for the immunohistochemical detection of lymphatic endothelial cells, and its presence is associated with lymphatic vessel caliber, primarily observed in capillaries comprising a single layer of endothelial cells, but not in larger lymphatic vessels containing smooth muscle cells [40,41]. Podoplanin expression was detected in all G1 and G2 rabbits and in one instance in G3. CD31, a transmembrane glycoprotein expressed in all continuous endothelial as well as in discontinuous endothelial cells of lymphatic vessels, macrophages, and platelets, was identified in all subjects. This glycoprotein plays a role in cell–cell adhesion between endothelial cells and between endothelial cells and lymphocytes [41]. The immunohistochemical staining outcomes align with the findings of the follow-up ICG examinations and histopathological analyses.

Our findings substantiate the capacity of the BioBridge^TM^ device to facilitate lymphangiogenesis and alleviate dermal backflow in rabbit hindlimbs, both in the initial preventive and treatment phases, and in a subsequent phase following the development of secondary lymphedema. Additionally, we confirmed that rabbits serve as a novel experimental animal model to study the lymphatic system through the induction of secondary lymphedema and the application of scaffolds that promote lymphangiogenesis.

This study was limited by the necessity of utilizing live animals, a factor that must be minimized when considering ethical concerns. An inherent limitation of this model is its inability to replicate the augmented hindlimb volume observed in secondary lymphedema as observed in human pathology. Additionally, the two evaluators were not blinded in this experimental study. Regarding the immunohistochemistry results, although the two markers used, CD31 and Podoplanin, effectively stained the endothelia, they also exhibited background reaction artifacts (nonspecific staining), potentially due to the secondary antibodies being generated in rabbits.

## 5. Conclusions

This study provides clear evidence supporting the efficacy of the BioBridge^TM^ device in promoting lymphangiogenesis in a rabbit model of secondary lymphedema induced by PLN excision and ILN lymphatic vessel sectioning using ICG lymphography. This is the first animal study to evaluate and demonstrate the efficacy of lymphedema treatment solely through BioBridge™ implantation. The consistent anatomy of the popliteal area in rabbits renders this model reproducible, facilitating a comprehensive investigation of the lymphatic system and its potential translational applications in human medicine.

## Data Availability

The raw data supporting the conclusions of this article will be made available by the authors on request.
